# Prediction of the SARS-CoV-2 (2019-nCoV) 3C-like protease (3CL ^pro^) structure: virtual screening reveals velpatasvir, ledipasvir, and other drug repurposing candidates

**DOI:** 10.12688/f1000research.22457.2

**Published:** 2020-04-09

**Authors:** Yu Wai Chen, Chin-Pang Bennu Yiu, Kwok-Yin Wong

**Affiliations:** 1 Department of Applied Biology & Chemical Technology, Hong Kong Polytechnic University, Hunghom, Hong Kong; 2 State Key Laboratory of Chemical Biology and Drug Discovery, Hunghom, Hong Kong; 3 Independent Researcher, La Costa, Ma On Shan, Hong Kong

**Keywords:** COVID-19, SARS, 2019-nCoV, 3C-like protease, drug repurpose, antiviral, coronavirus, virtual screening, molecular modelling, ledipasvir, velpatasvir, Hepatitis C virus, HCV

## Abstract

We prepared the three-dimensional model of the SARS-CoV-2 (aka 2019-nCoV) 3C-like protease (3CL ^pro^) using the crystal structure of the highly similar (96% identity) ortholog from the SARS-CoV. All residues involved in the catalysis, substrate binding and dimerisation are 100% conserved. Comparison of the polyprotein PP1AB sequences showed 86% identity. The 3C-like cleavage sites on the coronaviral polyproteins are highly conserved. Based on the near-identical substrate specificities and high sequence identities, we are of the opinion that some of the previous progress of specific inhibitors development for the SARS-CoV enzyme can be conferred on its SARS-CoV-2 counterpart.  With the 3CL ^pro^ molecular model, we performed virtual screening for purchasable drugs and proposed 16 candidates for consideration. Among these, the antivirals ledipasvir or velpatasvir are particularly attractive as therapeutics to combat the new coronavirus with minimal side effects, commonly fatigue and headache.  The drugs Epclusa (velpatasvir/sofosbuvir) and Harvoni (ledipasvir/sofosbuvir) could be very effective owing to their dual inhibitory actions on two viral enzymes.

## Introduction

On 7 January 2020, a new coronavirus, 2019-nCoV (now officially named SARS-CoV-2) was implicated in an alarming outbreak of a pneumonia-like illness COVID-19, originating from Wuhan City, Hubei, China. Human-to-human transmission was first confirmed in Guangdong, China ^1^. The World Health Organisation has declared this a global public health emergency — on 15 February 2020, there are more than 65,000 confirmed cases reported, and the death toll is over 1500. In the height of the crisis, this virus is spreading at a rate and scale far worse than previous coronaviral epidemics. By the time we finished revising this article (1 April 2020), it is a pandemic with more than 850,000 infected and total deaths of more than 42,000 affecting more than 180 countries/regions.

It was immediately evident from its genome that the coronavirus is evolutionarily related (80% identity) to the beta-coronavirus implicated in the severe acute respiratory syndrome (SARS), which originated in bats and was causative of a global outbreak in 2003. The momentum of research on developing antiviral agents against the SARS-CoV carried on after the epidemic subsided. Despite this, no SARS treatment has yet come to fruition; however, knowledge acquired from the extensive research and development efforts may be of use to inform the current therapeutic options.

The viral genome encodes more than 20 proteins, among which are two proteases (PL ^pro^ and 3CL ^pro^) that are vital to virus replication; they cleave the two translated polyproteins (PP1A and PP1AB) into individual functional components. The 3-chymotrypsin-like protease (3CL ^pro^, aka main protease, M ^pro^) is considered to be a promising drug target. Tremendous effort has been spent on studying this protein in order to identify therapeutics against the SARS-CoV in particular and other pathogenic coronaviruses (e.g. MERS-CoV, the Middle East respiratory syndrome coronavirus) in general because they share similar active sites and enzymatic mechanisms. The purpose of this study is to build a molecular model of the 3CL ^pro^ of the SARS-CoV-2 and to carry out virtual screening to identify readily usable therapeutics. It was not our intention, however, to comment on other structure-based drug design research as these will not be timely for the current epidemic.

## Methods

### Analysis of protein sequences

The translated polyprotein (PP1AB) sequence was obtained from the annotation of the GenBank entry of the SARS-CoV-2 genome (accession number MN908947). By comparing this sequence with the SARS-CoV PP1AB sequence (accession number ABI96956), the protease cleavage sites and all mature protein sequences were obtained. Sequence comparison and alignment were performed with BLASTp.

### Preparation of structural model

The high-resolution apo-enzyme structure of SARS-CoV 3CL ^pro^ (PDBID: 2DUC) ^2^ was employed as the template. The functional SARS-CoV 3CL ^pro^ is a dimer, therefore the SARS-CoV-2 enzyme was also constructed as a dimeric model, preserving all intermolecular interactions. The variant residues ( Table 1) were “mutated” *in silico* by SCWRL4 ^3^, followed by manual adjustment to ensure that the best side-chain rotamer was employed ( Table 2). The rebuilt model was subjected to steepest descent energy minimisation by Gromacs 2018.4 using the Gromos 54A7 forcefield, with a restraint force constant of 1000 kJ mol ^-1^ nm ^-2^ applied on all backbone atoms and all atoms of the vital residues ( Table 1). Accessible surface area of residues were calculated with *areaimol* of the CCP4 suite v7.0.

**Table 1.** Important residues of 3CL ^pro^ from SARS-CoV (conserved) and the SARS-CoV-2 variant residues. The residues that play functional roles in SARS-CoV 3CL ^pro^ are listed on the top three rows. These are absolutely conserved in the SARS-CoV-2 protein. The variant residues found in the SARS-CoV-2 protein are listed in the bottom row, with the SARS-CoV residues in brackets.

	Residue Number	Reference
**SARS-CoV**		
Catalytic	H41, C145	6
Substrate binding	H41, M49, G143, S144, 163–167, 187–192	2, 7
Dimerisation	R4, M6, S10, G11, E14, N28, S139, F140, S147, E166, E290, R298	8– 12
**SARS-CoV-2**		
Variant positions	V35(T), S46(A), N65(S), V86(L), K88(R), A94(S), F134(H), N180(K), V202(L), S267(A), A285(T), L286(I)	This work

**Table 2.** *In silico* mutagenesis to make the SARS-CoV-2 3CL ^pro^. The 12 variant residues with reference to the SARS-CoV enzyme are shown with the respective treatment of rotamer. “A” and “B” refers to the individual chains of the dimeric model. Both chains are in the crystal asymmetric unit and are not identical. The rotamer symbol (bracketed) is defined according to the conventions of Richardson ^13^, followed by its respective rank of popularity. ‘ASA’: accessible surface area (average of A and B chains) of the residue in the SARS-CoV 3CL ^pro^ structure, in Å ^2^ and in % relative to the ASA of a residue X in the Gly-X-Gly conformation.

Residue	Rotamer	ASA, Å ^2^ (%)	Remarks on replacement
T35V	AB: (t-), top	19 (14%)	conservative
A46S	A: (t-), 3rd; B: (p-), top	73 (63%)	A chain disordered, rotamer chosen to minimise steric clash
S65N	AB: (m-20), top	38 (28%)	
L86V	A: (m), 2nd; B: (t), top	0 (0%)	A chain rotamer to avoid clash
R88K	A: (mtpt), 9th; B: (mtpp), 19th	81 (33%)	AB: real-space refined with good fit to arginine densities
S94A	not applicable	64 (51%)	
H134F	AB: (m-85), top	57 (29%)	occupy similar but larger space
K180N	AB: (m-20), top	102 (50%)	
L202V	AB: (p), 3rd	22 (12%)	avoid steric clash
A267S	AB: (m), 2nd	0 (0%)	avoid steric clash
T285A	not applicable	68 (44%)	at dimeric interface
I286L	(mt), top	75 (46%)	at dimeric interface

### Virtual screening

MTiOpenScreen web service ^4^ was used for screening against its library of 7173 purchasable drugs (Drugs-lib), with 4574 unique compounds and their stereoisomers. Each library entry is identified with the name of the compound as well as an ZINC15 ID. The target binding site grid centre was specified by the active-site residues. At the MTiOpenScreen interface, the ‘Mode’ was set to ‘List of residues’ and these residues were specified: H41, M49, G143, S144, C145, H163, H164, M165, E166, L167, D187, R188, Q189, T190, A191 and Q192. The active sites on chain A and chain B, each derived from the catalytically-active dimeric model, were screened independently with AutoDock Vina ^5^.

When the crystal structure was released, it was stripped of its inhibitor and subjected to a screening.

The results returned from MTiOpenScreen is a list of 4500 target:ligand docking combinations (1500 ligands, each with 3 binding modes) ranked by binding energies. We listed the top 10 scorers of each chain as results. Stereoisomers of a compound (with the same drug name but unique ZINC15 IDs) that appear in the top list are collected together and presented as hits. The top ranking candidates for chains A and B were examined visually in PyMOL (version 1.7.X) ^14^.

An earlier version of this article can be found on ChemRxiv (DOI: 10.26434/chemrxiv.11831103.v2).

## Results

### High sequence homology with SARS-CoV

The first available genome was GenBank MN908947, now NCBI Reference Sequence NC_045512. From it, the PP1AB sequence of SARS-CoV-2 was extracted and aligned with that of SARS-CoV. The overall amino-acid sequence identity is very high (86%). The conservation is noticeable at the polyprotein cleavage sites. All 11 3CL ^pro^ sites ^2^ are highly conserved or identical ( *Extended data* ^15^, Table S1), inferring that their respective proteases have very similar specificities. The 3CL ^pro^ sequence of SARS-CoV-2 has only 12 out of 306 residues different from that of SARS-CoV (identity = 96%).

### Conserved sequence identity among SARS-CoV-2

We compared the polyprotein PP1AB and the 3CL ^pro^ sequences among all 11 SARS-CoV-2 genomes (GenBank MN908947, MN938384, MN975262, MN985325, MN988668, MN988669, MN988713, MN994467, MN994468, MN996527 and MN996528) that were available on 1 February 2020. With reference to MN908947 (NC_045512), among the 7096 residues, there is only one variable residue in each of MN975262 (in NSP-4), MN994467 (in NSP-2), MN994468 (in NSP-13), MN996527 (in NSP-16); and two in MN988713 (in NSP-1 and NSP-3). The remaining five have no difference. To summarise, all SARS-CoV-2 3CL ^pro^ sequences and all their cleavage junctions on their polyproteins are 100% conserved.

### 3D model of the SARS-CoV-2 3CL ^pro^

The amino acids that are known to be important for the enzyme’s functions are listed in Table 1. Not unexpectedly, none of the 12 variant positions are involved in major roles. Therefore, we are confident to prepare a structural model of the SARS-CoV-2 3CL ^pro^ by molecular modelling ( *Extended data* ^15^, Figure S1), which will be immediately useful for *in silico* development of targeted treatment. After we submitted the first draft of this study, the crystal structure of SARS-CoV-2 3CL ^pro^ was solved and released (PDB ID 6LU7) ^16^, which confirms that the predicted model is good within experimental errors ( *Extended data* ^15^, Figure S2).

### Virtual screening for readily available drugs

The list of 1500 results has Autodock Vina binding energies ranging from -10.1 to -7.6 (mean = -8.2) kcal mol ^-1^ from chain A active site; and -8.7 to -6.5 (mean = -7.1) kcal mol ^-1^ for that of chain B. When examined in molecular graphics ^14^, all solutions were found to fit into their respective active sites convincingly. The binding energies of chain A complexes were generally higher than those of chain B by approximately 1.4 kcal mol ^-1^ among the top scorers ( Table 3). This presumably demonstrates the intrinsic conformational variability between the A- and B-chain active sites in the crystal structure (the average root-mean-square deviation (rmsd) in Cα atomic positions of active-site residues is 0.83 Å). In each screen, the differences in binding energies are small, suggesting that the ranking is not discriminatory, and all top scorers should be examined. We combined the two screens, merged stereoisomers, and found 16 candidates which give promising binding models (etoposide and its phosphate counted as one) ( Table 3). One drug (dirlotapide) which is not intended for human use was excluded. All possible isomers of compounds with multiple stereoisomers are found in the full screening results of 1500, in particular: 38 of hesperidin, 34 of teniposide, 32 of etoposide and 21 of etoposide-phosphate.

**Table 3.** The results of virtual screening of drugs on the active sites of SARS-CoV-2 3CL ^pro^ model. The left and right columns are the results of A and B chains, respectively. The top scorers are listed first, then the equivalent top scorers of the other chain listed at the lower half. ‘M.W.’: molecular weight in g mol ^-1^. ‘B.E.’: AutoDock Vina binding energy in kcal mol ^-1^. The ‘Hits’ column is the number of times a compound appears as top scorers (representing different stereoisomers) out of the total number of stereoisomers of that compound in the library; only the binding energy of the top-ranking hit was shown. Etoposide and its phosphate are listed separately in the screens. ‘n.f.’ = not found. Approved and pre-approved drugs are shown in green and orange, respectively. Except dihydroergocristine and ditercalinium, all approved drugs have undergone post-market surveillance, i.e. Phase 4. The mean score of each screen (1500 results), scores of lopinavir and ritonavir are included at the bottom for reference.

A Chain				B Chain			
A Top scorers	M.W.	B.E.	Hits	B Top scorers	M.W.	B.E.	Hits
diosmin	609	-10.1	1/1	etoposide	669	-8.7	1/32
hesperidin	611	-10.1	8/38	R428	507	-8.6	2/2
MK-3207	558	-10.1	1/4	MK-3207	558	-8.6	1/4
venetoclax	868	-10.0	1/1	teniposide	657	-8.5	2/34
dihydroergocristine	612	-9.8	1/6	UK-432097	778	-8.5	1/2
bolazine	-9.8	-9.8	1/1	eluxadoline	570	-8.4	1/1
R428	507	-9.8	2/2	venetoclax	868	-8.4	1/1
ditercalinium	719	-9.8	1/1	ledipasvir	889	-8.4	1/1
etoposide-phosphate	669	-9.8	1/21	irinotecan	587	-8.4	1/1
				lumacaftor	452	-8.4	1/1
				velpatasvir	883	-8.4	1/5
(B Top scorers)				(A Top scorers)			
teniposide		-9.7		hesperidin		-8.3	
etoposide		-9.7		etoposide-phosphate		-8.3	
UK-432097		-9.6		bolazine		-8.3	
irinotecan		-9.5		dihydroergocristine		-8.1	
lumacaftor		-8.9		diosmin		-7.9	
velpatasvir		-8.5		ditercalinium		-7.7	
eluxadoline		-8.0					
ledipasvir		n.f.					
(Reference)				(Reference)			
Mean of 1500		-8.2		Mean of 1500		-7.1	
lopinavir		-8.0		lopinavir		-6.8	
ritonavir		-7.9		ritonavir		-6.9	

### Assessment of the candidate drugs

**Figure 1.**
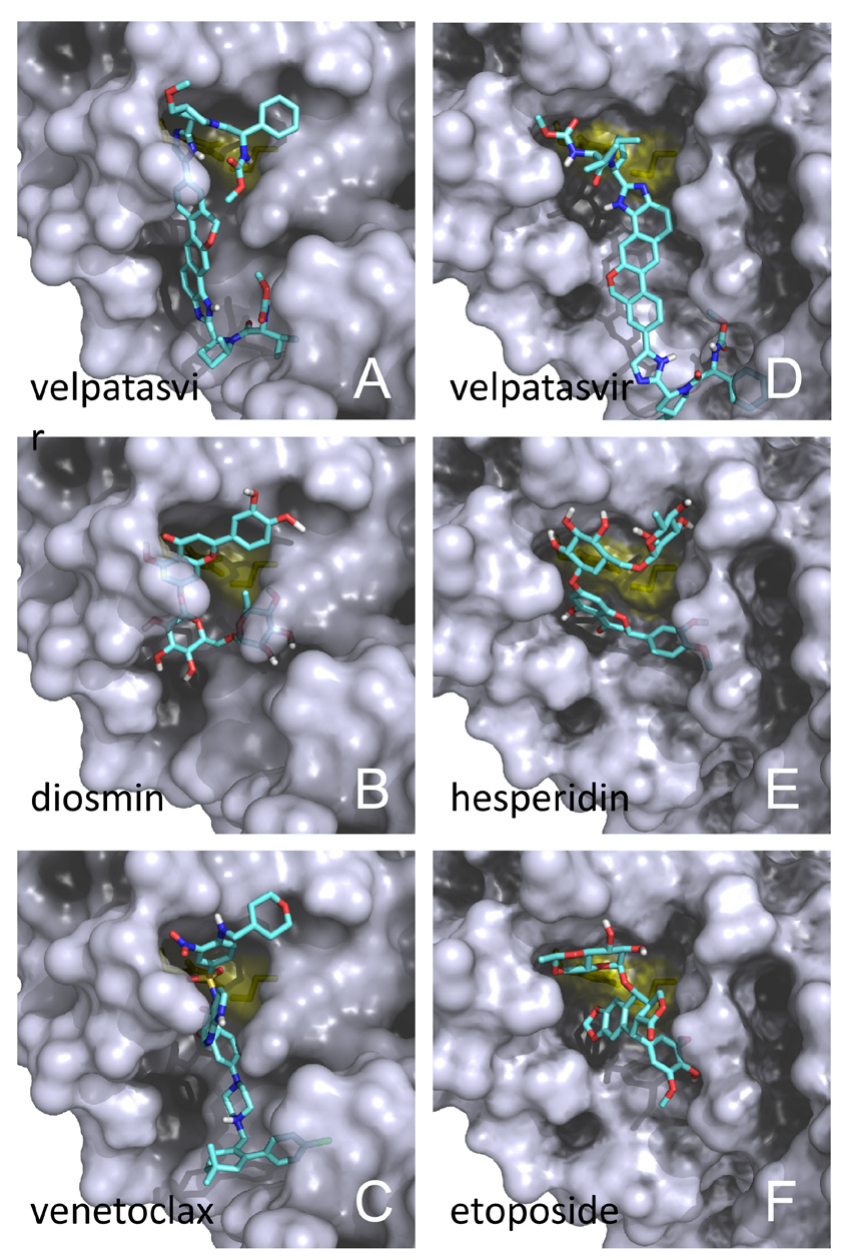
Virtual screening results for the SARS-CoV-2 3CL ^pro^ protease. Docking of representative drugs into the active sites of A chain ( **A**, **B**, **C**) and that of B chain ( **D**, **E**, **F**). The catalytic residue surfaces are coloured in yellow. Atom colours of drug: C: cyan; O: red; N: blue; H: white; S: yellow; only polar hydrogens are shown. Prepared with PyMOL.

We checked the actions, targets and side effects of the 16 candidates. Among these, we first noticed velpatasvir ( Figure 1A, Figure 1D) and ledipasvir, which are inhibitors of the NS5A protein of the hepatitis C virus (HCV). Both are marketed as approved drugs in combination with sofosbuvir, which is a prodrug nucleotide analogue inhibitor of RNA-dependent RNA polymerase (RdRp, or NS5B). Interestingly, sofosbuvir has recently been proposed as an antiviral for the SARS-CoV-2 based on the similarity between the replication mechanisms of the HCV and the coronaviruses ^17^. Our results further strengthen that these dual-component HCV drugs, Epclusa (velpatasvir/sofosbuvir) and Harvoni (ledipasvir/sofosbuvir), may be attractive candidates to repurpose because they may inhibit two coronaviral enzymes. A drug that can target two viral proteins substantially reduces the ability of the virus to develop resistance. These direct-acting antiviral drugs are also associated with very minimal side effects and are conveniently orally administered ( Table 4). These computational results provide a rationale for experimental validation of inhibiting the SARS-CoV-2 with velpatasvir and ledipasvir, which is underway.

**Table 4.** Possible side effects and routes of administration of the drugs identified from virtual screening for SARS-CoV-2 3CL ^pro^.

Drug	Possible side effects (adverse reactions)	Admin.
Diosmin ^a, b^	Mild gastrointestinal disorders; skin irritations; nausea; heart arrhythmias	Topical; oral
Hesperidin ^a, d^	Stomach pain and upset; diarrhea; headache	Oral
MK-3207 ^c^	No information	Oral
Venetoclax ^a, b^	Neutropenia; nausea; anaemia, diarrhea; upper respiratory tract infection	Oral
Dihydroergocristine ^a^	No information	Oral
Bolazine ^b^	No information	Intramuscular
R428 ^b^	No information	Oral
Ditercalinium	No information	No info
Etoposide ^a, b^	Alopecia; constipation; diarrhea; nausea; vomiting; secondary malignancies	Intravenous
Teniposide ^a, b^	Gastrointestinal toxicity; hypersensitivity reactions; reversible alopecia	Intravenous
UK-432097 ^c^	No information	Inhaled
Irinotecan ^a, b^	Gastrointestinal complication	Intravenous
Lumacaftor ^a^	Dyspnea; nasopharyngitis; nausea; diarrhea; upper respiratory tract infection	Oral
Velpatasvir ^a, b^	Headache; fatigue; nausea	Oral
Eluxadoline ^a, b^	Constipation; nausea; fatigue, bronchitis, viral gastroenteritis; pancreatitis	Oral
Ledipasvir ^a^	Fatigue; headache	Oral

Sources of information: ^a^ DrugBank.ca (main), ^b^ Wikipedia.org, ^c^ ClinicalTrials.gov and ^d ^WebMD.com.

The flavonoid glycosides diosmin ( Figure 1B) and hesperidin ( Figure 1E), obtained from citrus fruits, fit very well into and block the substrate binding site. Yet, these compounds cause mild adverse reactions ( Table 4). Hesperidin has 38 stereoisomeric forms and several of these showed up among the top scorers ( Table 3; Figure 1E). It has been reported to be a good inhibitor of the SARS-CoV 3CL ^pro^ with an IC _50_ of 8.3 µM in a cell-based assay ^18^.

Teniposide and etoposide (and its phosphate) are chemically related and exhibited good binding models ( Figure 1F). However, these chemotherapy drugs have a lot of strong side effects and need intravenous administration ( Table 4). The approved drug venetoclax ( Figure 1C) and investigational drugs MK-3207 and R428 scored well in both screens. Venetoclax is another chemotherapy drug that is burdened by side effects including upper respiratory tract infection ( Table 4). Not much has been disclosed about MK-3207 and R428.

We subjected the crystal structure to the same virtual screening procedures. A very similar list of candidates showed up consistently ( *Extended data* ^15^, Table S2) with high scores although ledipasvir was not found.

We noticed that most of the compounds on the list have molecular weights (MW) over 500 ( Table 3), except lumacaftor (MW=452). The largest one is ledipasvir (MW=889). This is because the size of the peptide substrate and the deeply buried protease active site demand a large molecule that has many rotatable dynamics to fit into it.

## Discussion

We identified five trials on ClinicalTrials.gov involving antiviral and immunomodulatory drug treatments for SARS ( Table 5), all without reported results; i.e., at present, there are no safe and effective drug candidates against SARS-CoV. This is because once the epidemic is over, there are no patients to recruit for clinical trials. Only the study with streptokinase succeeded in completion of phase 3. It is disappointing that little progress in SARS drug development has been made in the past 17 years. After the 2003 outbreak, numerous inhibitors for the 3CL ^pro^ enzyme have been proposed ^19, 20^, yet no new drug candidates have succeeded to enter the clinical phase 1.

**Table 5.** Drugs targeting SARS that are registered for the U.S. Food and Drug Administration (USFDA) clinical trials (as of mid-February 2020). ‘n.i.’ = no information.

Drug	Condition	Phase	Status	From	To	Location
Lopinavir / Ritonavir + Ribavirin	SARS	Unknown	Unknown	n.i.	n.i.	Hong Kong
Alferon LDO	SARS	Phase 2	Completed	Nov 04	Apr 06	Hong Kong
Poly-ICLC	Respiratory viruses ^a^	Phase 1	Completed	Mar 08	Dec 09	USA
Streptokinase vs. Heparin	SARS, ARDS	Phase 3	Completed	Feb 16	Jan 18	n.i.
Glucocorticoid (methylprednisolone) therapy	Coronavirus infections ^b^	Phase 2, Phase 3	Unknown	Jan 20	Dec 20 (Est.)	China

^a^ This covers unknown respiratory viruses. ^b^ This includes the COVID-19. ‘Est.’ = estimated. ‘ARDS’ = acute respiratory distress syndrome.

One record which receives a lot of attention amid the current outbreak is the lopinavir/ritonavir combination ^21^. They are protease inhibitors originally developed against HIV. During the 2003 SARS outbreak, despite lacking a clinical trial, they were tried as an emergency measure and found to offer improved clinical outcome ^21^. However, some scientists did express scepticism ^22^. By analogy, these compounds were speculated to act on SARS-CoV 3CL ^pro^ specifically, but there is as yet no crystal structure to support that, although docking studies were carried out to propose various binding modes ^23– 26^. The IC _50_ value of lopinavir is 50 μM ( *K*_i_ = 14 μM) and that for ritonavir cannot be established ^27^. These two compounds turned up in our virtual screening results, with scores slightly lower than the mean scores ( Table 3). Based on our results that the two CoV 3CL ^pro^ enzymes are identical as far as active sites and substrate specificities are concerned, we were of the opinion that it was still one of the recommended routes for immediate treatment at the time of writing the first version (mid-February 2020). Disappointedly, the latest trial of lopinavir/ritonavir on COVID-19 showed no clinical benefit ^28^.

If we look beyond the 3CL ^pro^, an earlier screen produced 27 candidates that could be repurposed against both SARS-CoV and MERS-CoV ^29^. In addition, the other coronaviral proteins could be targeted for screening. Treatment of the COVID-19 with remdesivir (a repurposed drug in development targeting the RdRp) showing improved clinical outcome has earlier been reported and clinical trial is now underway ^30^.

We consider this work part of the global efforts responding in a timely fashion to fight this deadly communicable disease. We are aware that there are similar modelling, screening and repurposing exercises targeting 3CL ^pro^ reported or announced ^23, 31– 37^ (up to mid-February 2020). Our methods did not overlap, and we share no common results with these studies. During revision, another crystal structure paper was published ^38^.

## Data availability

### Source data

The 11 SARS-CoV-2 polyprotein PP1AB and 3CL ^pro^ sequences used in this study were obtained from NCBI GenBank, accession numbers MN908947 , MN938384, MN975262, MN985325, MN988668, MN988669, MN988713, MN994467, MN994468, MN996527 and MN996528, available on 1 February 2020.

The SARS-CoV PP1AB sequence was obtained from NCBI Protein, accession number ABI96956.

The two coronavirus protease structures used were obtained from Protein Data Bank, ID 2DUC and 6LU7.

### Extended data

Open Science Framework: SARS-CoV-2 (2019-nCoV) 3CLpro Model and Screening. https://doi.org/10.17605/OSF.IO/FD243 ^15^.

The “Virtual Screening” folder contains the following extended data:

- 2019-nCoV-3CLpro.pdb. (3D model of the 3CL ^pro^: A and B chains.)
- A-screen4500.pdbqt, B-screen4500.pdbqt, X-screen4500.pdbqt. (Virtual screening 3D results of Model A chain, Model B chain and the crystal-structure (A chain) in PDBQT format (can be viewed by any text editor). Use the software PyMOL to open these files. Each result file contains 4500 drug-to-protein docking hits ranked by AutoDock Vina binding energies in kcal mol ^-1^.)
- A-screen1500.table.csv, B-screen1500.table.csv, X-screen1500.table.csv. (Virtual screening results (names only) of Model A chain, Model B chain and the crystal-structure (A chain) in CSV format (can be opened by Excel or any text editor). This is a summary of the top 1500 drug-to-protein docking hits ranked by AutoDock Vina binding energies in kcal mol ^-1^.)

The “Extended Results” folder contains the following extended data:

- Tab S1.docx (Sequence homology of the 3CL ^pro^ cleavage junctions of PP1AB between SARS-CoV-2 and SARS-CoV).
- Tab S2-v2.docx (The results of virtual screening of drugs on the active site of SARS-CoV-2 3CL ^pro^ crystal structure).
- Fig S1.pptx (The structural model of the SARS-CoV-2 3CL ^pro^ protease).
- Compare Crystal.docx (A comparison, with Figure S2, of the active sites of model chains A, B and the crystal structure).

Data are available under the terms of the Creative Commons Zero “No rights reserved” data waiver (CC0 1.0 Public domain dedication).
