# Supplementary material for: Prediction of the SARS-CoV-2 (2019-nCoV) 3C-like protease (3CL pro) structure: virtual screening reveals velpatasvir, ledipasvir, and other drug repurposing candidates
Source: F1000Res. 2020 Apr 9;9:129. Originally published 2020 Feb 21. [Version 2] doi: 10.12688/f1000research.22457.2 (PMC7062204; doi:10.12688/f1000research.22457.2)
Supplement: Peer review history

# Peer review: Reviewer response for version 1

**DOI:** 10.5256/f1000research.24784.r60415

**Published:** 2020-03-17

**Authors:** Vip Viprakasit, Warut Tulalamba

**Affiliations:** 1 Division of Pediatric Haematology and Oncology, Thalassemia Center, Department of Pediatrics, Faculty of Medicine Siriraj Hospital, Mahidol University, Bangkok, Thailand; 2 Research Division and Thalassemia Center, Faculty of Medicine Siriraj Hospital, Mahidol University, Bangkok, Thailand

In this article, Chen YW and his colleagues carried out virtual screening using the computational molecular modeling of the viral protein from SARS-CoV-2 or COVID-19 to identify therapeutics targets. The authors presented that 3CLpro protease enzyme of the SARS-CoV-2 is considered to be a promising drug target and repurposing accessible drugs to challenge the globally outbreaking of the SARS-CoV-2. The authors initially used the translated polyprotein (PP1AB) sequence of SARS-CoV-2 and SARS-CoV to prepare for protein structural model. Subsequently, they performed computational virtual screening against this against its using a library of purchasable drugs with the binding site grid. Among > 7,000 repurposing drugs in the screening with their known side effects, the antivirals ledipasir or velpatasvir are potentially used against SARS-CoV-2 infection with minimal side effects. The manuscript is straightforward in both terminology and structure. The manuscript can be considered to be accepted with a minor revision and could be further improved with following points:

1. Table 2 is mentioned prior Table 1 in the manuscript.
2. More details of the setting and cut-off used in the virtual screening and analysis should be provided in the Method section.
3. Table 1 is quite confusing. The importance residues of the SARS-CoV 3CLpro previously reported and the variant residues found in SARS-CoV-2 (this work) should be separated. The amino acid variants of each position should be included. Using the image with the annotation could be an alternative and more informative presentation.
4. In Table 3, some information should be included in the table, such as molecular weight. In addition, the authors should discuss more about the results shown in the Table 3 to compare the binding energy different between A and B chains.
5. In the conclusion, the authors proposed velpatasvir and ledipasvir as an attractive candidate. However, based on the virtual screening on the active sites of SARS-CoV-2 3CLpro model, both of them are not ranked as the top list in Chain A screening. Could you please explain this scenario? The results from other virtual screening package (such as Glide or FlexX) should be compared?
6. To extend the interest of the topic as well as to compare the potential for using repurposing drug in COVID-19 treatment, the drug virtual screening with other viral enzymes might be performed and compared. In this case, since there are several clinical researches for using this drug family (e.g. Lopinavir/ritonavir) in COVID-19 treatment, therefore, the authors can compare the virtual screening model with the clinical outcomes.

We confirm that we have read this submission and believe that we have an appropriate level of expertise to confirm that it is of an acceptable scientific standard.

# Response

**Published:** 2020-04-01

**Authors:** Yu Wai Chen

**Affiliations:** Hong Kong Polytechnic University, Hong Kong

**1. **This is now corrected.  

**2. **In reporting our virtual screening results, we listed the top 10 scorers of each model. We employed a ‘soft’ cutoff at which all results having the same binding energy were included.  This led to 10 (one non-human vet drug later excluded) and 11 results in chains A and B, respectively.  This is now added to Methods:VS.  We revised the Methods:VS section to make the procedures in MTiOpenScreen clearer.

**3.** **Table 1** is revised so that important residues in SARS-CoV and variant residues in SARS-CoV-2 are now separated.  The amino acids are identified with their single-letter codes.

We keep the two lists together because the reader can immediately compare them and see that the two sets do not overlap.  The same information is conveyed in 3D on **Fig. S1** in *Extended data*.

**4.** Molecular weights were added accordingly.  The ‘hits’ column was revised to represent the number of stereoisomers found, out of the total possible numbers. **Table 3** caption was also revised to explain in detail.

The two chains are structurally non-identical (rmsd in Cα=0.83Å) in the dimer and in the crystal structure.  The differences lie primarily in side-chain conformations, especially for residues with long side chains.  Thus, the two chains are two slightly-different conformational states of the protein.  It is advantageous to have two states for VS because it allows some degrees of conformational variability of the active sites to be taken into account.  AutoDock Vina implemented in MTiOpenScreen does not allow for the flexibility of the active site.  Therefore, one would not expect the active sites on the two chains to yield the same results in terms of binding energies and ligand ranking.  Further, the AutoDock Vina 'binding energy' is not a true binding free energy *per se* but an analogous empirical scoring function for the sake of assessment.  We interpreted these results semi-quantitatively to extract trends, in VS results section, instead of exercising detailed energetic comparisons. 

**5. **In VS results, we identified 16 candidates. Our first intention was to present all these to the readers as top scorers of docking ranked by AutoDock Vina binding energies.  Next, in the Assessment section, we discussed the known properties of some of these which stood out, also considering their side effects.  We highlighted velpatasvir and ledipasvir mainly because of their minimal side effects, which is a crucial factor in repurposing.  In addition, we noticed the two drugs which correspond to these two compounds also contain sofosbuvir which was identified as an anti-SARS-CoV-2 candidate in a separate study. It was because of all these factors, velpatasvir and ledipasvir stood out among the 16 candidates.  Therefore we highlighted them in the conclusion.  We also mentioned other top-ranking candidates but they may have strong side effects.  We briefly assessed these candidates to inform the clinicians who may be interested in these results.

We did not intend for a comprehensive study comparing with other VS packages.  Here we employed AutoDock Vina, one of the most popular VS utilities, and produced results for follow-up development. We are moving quickly to the next stage of experimental verification in order to respond to the rapidly worsening global crisis.

**6. **This work was intended to be a fast response to the current pandemic — thus, we focused on one of the several potential targets for antiviral development.  To this day, there are several parallel efforts on this and other targets at different scales.  At the time we revised it (end of March, 2020), this article is outdated in some aspects.  Therefore, we shall not extend the work further on the computational methods, as this is not the most pressing.

As the reviewer mentioned, lopinavir and ritonavir have been clinically tried.  These two compounds are also expected to be specific inhibitors of 3CL ^pro^. Therefore, we checked to see if VS picked up these two drugs: indeed it did, with a medium score (now included in **Table 3**) quite a bit lower than the top candidates.  Docking of lopinavir/ritonavir into the SARS-CoV 3CL ^pro^ has been performed previously (referenced in the main text) and our models have basically the same active sites.  Disappointedly, the latest trial of lopinavir/ritonavir on COVID-19 showed no clinical benefit.  We added a sentence in Discussion to update the readers about this clinical trial.

# Peer review: Reviewer response for version 1

**DOI:** 10.5256/f1000research.24784.r60414

**Published:** 2020-03-06

**Authors:** Wai-Lung Ng

**Affiliations:** 1 School of Pharmacy, Faculty of Medicine, The Chinese University of Hong Kong (CUHK), Hong Kong, Hong Kong

Since December 2019, a plethora of cases resembling viral pneumonia has emerged exponentially in Wuhan, China, which is now coined Coronavirus Disease 2019 (COVID-19; formerly known as 2019-nCoV).

 Chen *et. al.* reported the computational modelling and virtual screening results of the 3C-like protease (3CLpro) of SARS-CoV-2. This study is timely in view of the recent outbreak of COVID-19. The rationale of repurposing existing drugs to tackle the global viral outbreak is sound. The manuscript is also well-written and structured. It should be noted that:

- The authors compared their model with the recently published crystal structure of 3CLpro and found a high similarity between the two structures. They also obtained a similar list of top-ranked drug candidates when the crystal structure was subjected to the same screening protocol.
- Several studies using similar modeling and virtual screening approaches have also been published recently. 

Some suggestions for improving the manuscript:

- The authors proposed that the HCV drugs velpatasvir and ledipasvir, and thus Epclusa and Harvoni, could be attractive drug candidates for treating SARS-CoV-2 infection. However, there is no direct evidence to support this claim. To support this claim, the authors should connect the computational results with experimental data. To test their hypothesis, the authors should at least prove (or disprove) that the two HCV drugs could inhibit the biochemical activity of 3CLpro of SARS-CoV-2.
- To further test the hypothesis, the two NS5A inhibitors should be tested using in vitro assays such as viral RNA PCR assay.
- If there are no such experimental data to support the claim, the authors may consider revising their conclusion to "the computational results provide a rationale for further experimental validation of treating SARS-CoV-2 with velpatasvir and ledipasvir".

I confirm that I have read this submission and believe that I have an appropriate level of expertise to confirm that it is of an acceptable scientific standard.

# Response

**Published:** 2020-04-01

**Authors:** Yu Wai Chen

**Affiliations:** Hong Kong Polytechnic University, Hong Kong

**1. **Experimental work to test these results is underway.  This future plan is now included in the last sentence of the section Results:Assessment of the candidate drugs.

**2.** We agree that it is important to also study the effects of the two NS5A inhibitors.  We plan to set up collaborations for that purpose.

**3.** This is related to point number 1. A sentence was added at the end of the section Results:Assessment of the candidate drugs to this effect.

# Peer review: Reviewer response for version 1

**DOI:** 10.5256/f1000research.24784.r60688

**Published:** 2020-03-06

**Authors:** Jing Huang, Qiaozhu Tan

**Affiliations:** 1 School of Life Sciences, Westlake University, Hangzhou, China

Yu Wai Chen and co-workers presented a molecular modeling and docking study of the 3CL protease in the SARS-CoV-2 virus. The manuscript started with comparing polyprotein PP1AB sequences of SARS-CoV-2 and SARS-CoV, based on which the 3D structure of SARS-CoV-2 3CLPro protein was constructed. The authors then performed virtual screening against SARS-CoV-2 3CLPro using a library of 7173 purchasable drugs. Considering both binding affinities and known side effects, the authors recommend velpatasvir and ledipasvir, and further suggest combining them with another HCV RdRp inhibitor sofosbuvir, aka repurposing the Epclusa and Harvoni for treating the coronavirus. This is a concise and timely report, and has proposed new therapeutic possibilities for the SARS-CoV-2 virus. The manuscript could be further improved by addressing the following comments.

1. More details of the docking should be provided. What's the binding energy cutoff used? How is the hits (reported in Table 3) used? 3CLpro is catalytically active as a dimer. How is this considered in the virtual screening? What does the "(B Top scorers)" mean?
2. In the extended data of virtual screening, one compound could have multiple entries with different ZINC numbers. For example hesperidin corresponds to at least 20 different compounds. What are the difference? And how are different results assembled?
3. Table 1 is not clear. Please do a column-by-column comparison between different sites of SARS-CoV and SARS-CoV-2. Also please add one-letter amino acid codes for the residues.
4. The constructed protein structure is very similar to the recently solved crystal structure (6LU7), as "... confirms that the predicted model is good within experimental errors", but the docking results seem to differ significantly. Could the authors explain?

We confirm that we have read this submission and believe that we have an appropriate level of expertise to confirm that it is of an acceptable scientific standard.

# Response

**Published:** 2020-04-01

**Authors:** Yu Wai Chen

**Affiliations:** Hong Kong Polytechnic University, Hong Kong

**1. **In reporting our virtual screening results, we listed the top 10 scorers of each model. This is now added to Methods:VS.

 The ‘hits’ is the number of times a compound appears (representing different stereoisomers) out of the total number of possible stereoisomers of that compound in the library.  For example, hesperidin has a large number of stereoisomers (38) in the library and 8 of these are among the top scorers, it can give some confidence of reproducibility of the screening method.  Stereoisomers are expected to have different binding energies.  This is clarified now in the Table 3 caption.

 The functional SARS-CoV 3CL ^pro^ is a dimer. Therefore the SARS-CoV-2 enzyme was also constructed as a dimeric model, preserving all intermolecular interactions.  The A- and B-chain active sites were screened independently but each was derived from the catalytically-active dimeric model.  This information was now included in the Methods: Preparation of structural model and the VS sections.

 The “(B Top scorers)” in the A column of Table 3 refers to those compounds which are top scorers of the B site but did not turn up among the A site top 10; they were listed to show that they still scored high (compared to the mean) although not at the top.  This was explained in the Table caption.

**2.** The drugs-lib has 7173 purchasable drugs, with 4,574 unique compounds and their stereoisomers.  Each entry is identified with the name of the compound as well as a ZINC15 ID.  Stereoisomers of a compound (with same drug name but unique ZINC15 IDs) are collected together and presented as hits out of total possible stereoisomers.  These are now described in Methods: VS and the Table 3 caption.

**3. **The purpose of Table 1 is to show that the variant positions do not overlap with any of the functional residues.  It is not meant to be a detailed comparison. We amended the table to include one-letter residue identities. The list of dimerisation residues was revised to include R4, M6, E166 and E290. One reference was added.

**4.** The crystal structure virtual screening results (Table S2) is consistent with those of the models.  Of the 9 top scorers, 6 were in common with the model top scorers (top half of table: Top scorers).  For the remaining 10 of the model candidates, all of them were found in the full-list results of the crystal structure screen (bottom half of table: A, B model scorers), all with higher or similar scores to the two reference compounds, lopinavir and ritonavir.  Given the semi-quantitative nature of VS and binding energy scoring using a static active site, these results from slightly different active-site conformations are considered to be agreeable.
